# Supplementary material for: Chronic Exposure to TDI Induces Cell Migration and Invasion via TGF-β1 Signal Transduction
Source: Int J Mol Sci. 2023 Mar 24;24(7):6157. doi: 10.3390/ijms24076157 (PMC10093867; doi:10.3390/ijms24076157)
Supplement: Supplementary file 1 [file ijms-24-06157-s001.zip › ijms-2270709-supplementary.pdf]

Supplementary Table S1. Total RNA quality used for mRNA quantification sequencing.

Sample	ng/µl	Total (µg)	OD value 260/280	OD value 260/230	Ratio 28s/18s	RIN
**Con**	436.5	13.967	2.05	2.14	1.9	9.7
**TDI50**	349.8	11.194	2.08	1.89	2.0	9.7
**TDI250**	310.0	9.920	2.11	2.02	2.2	9.7

Supplementary Table S2. Primer sequence used for qRT-PCR.

Genes	Forward primers	Reverse primers	Product size
Vimentin (*homo sapiens*)	5′-AGGCAAAGCAGGAGTCCACTGA-3′	5′-ATCTGGCGTTCCAGGGACTCAT-3′	100bp
Fibronectin (*homo sapiens*)	5′-ACAACACCGAGGTGACTGAGAC-3′	5′-GGACACAACGATGCTTCCTGAG-3′	143bp
α-SMA (*homo sapiens*)	5′-CCATCGTCCACCGCAAAT-3′	5′-TGAGAAGATTCGTCGTCCTG-3′	75bp
E-cadherin (*homo sapiens*)	5′-TATTGAAAGAGAAACAGGATGG-3′	5′-CGTGAGAGAAGAGAGTGTAT-3′	83bp
N-cadherin (*homo sapiens*)	5′-CCTCCAGAGTTTACTGCCATGAC-3′	5′-GTAGGATCTCCGCCACTGATTC-3′	149bp
TGF-β1 (*homo sapiens*)	5′-CTATGACAAGTTCAAGCAGAG-3′	5′-GAGGTATCGCCAGGAATT-3′	187bp
TGFBR1 (*homo sapiens*)	5′-GTGCCTTGAGTTGGTGTA-3′	5′-ATGGGATAGTGATTTTGTTGTG-3′	195bp
TGFBR2 (*homo sapiens*)	5′-TGAAGAACGACCTAACCT-3′	5′-TCATCCACAGACAGAGTAG-3′	76bp
GAPDH (*homo sapiens*)	5′-TATGACAACAGCCTCAAGAT-3′	5′-GAGTCCTTCCACGATACC-3′	105bp

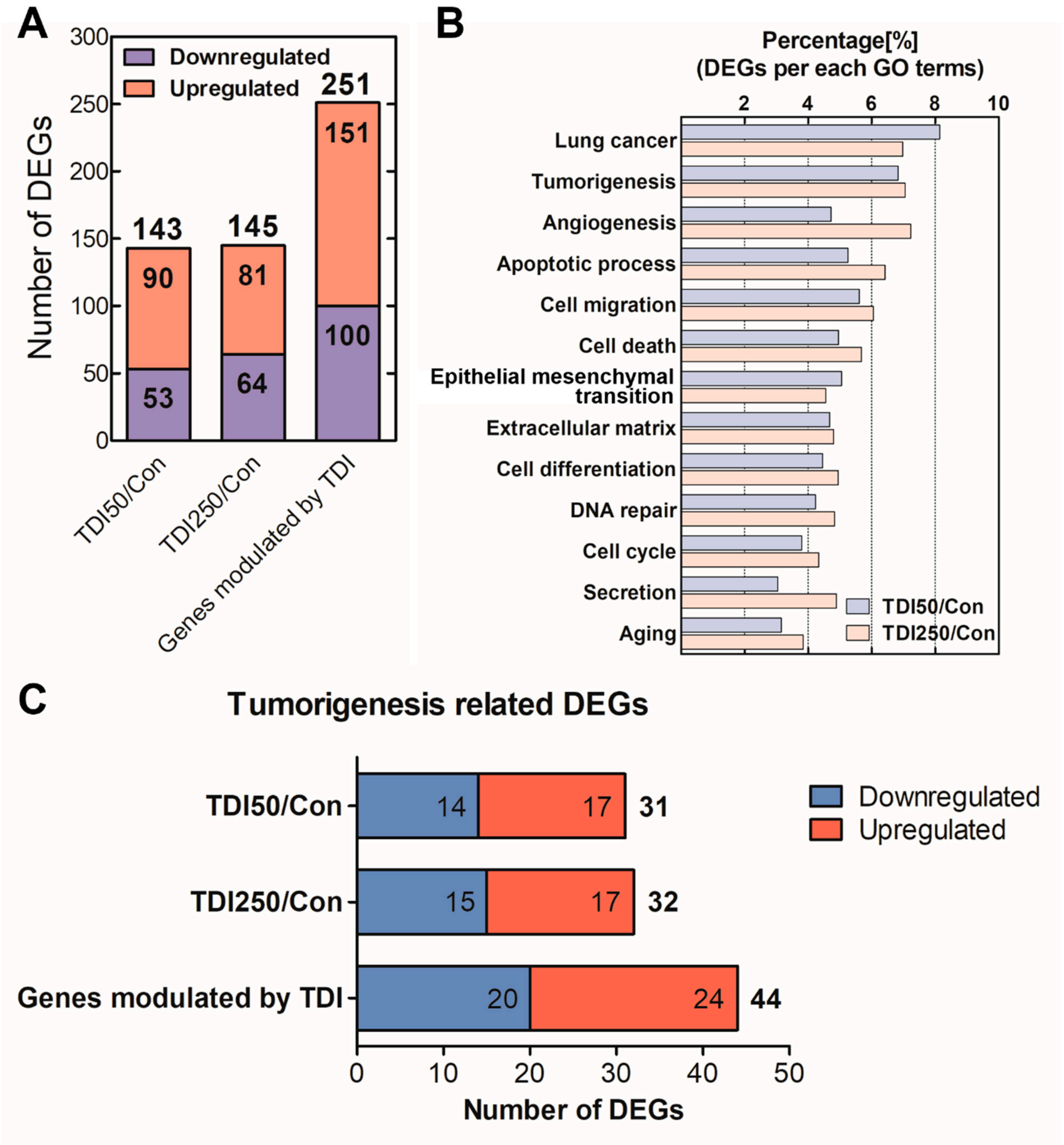

Supplementary Figure S1. Selection of gene modulated by TDI exposure in BEAS-2B

(A) After exposure to 50 μM and 250 μM of TDI for 24 hours, we selected the differentially expressed genes (DEGs) whose expression values were modified by ≥2 folds compared to the control, and a $\log_2$-normalized value of genes were 5 or over to minimize false counts. (B) Gene ontology analysis was conducted using 251 DEGs modulated by TDI. (C) Among genes related to tumorigenesis, 44 DEGs were significantly modulated by TDI. The expression of 44 DEGs were altered by ≥1.4 folds compared to the control, and a $\log_2$-normalized value of genes was 5 or over to minimize false counts. TDI250: 250 μM of TDI; TDI50: 50 μM of TDI; Con: control.

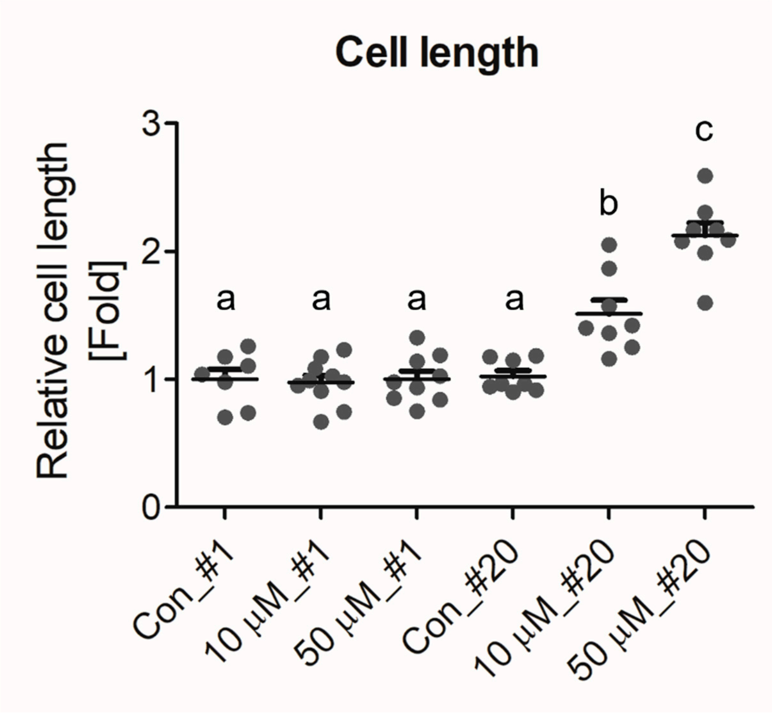

Supplementary Figure S2. Cell length altered by chronic TDI exposure

The relative cell length of the TDI-exposed cell was represented through the plot. Cell lengths were measured through ImageJ using more than ten cells. a, b, and c represent groups with the statistically same tendency, respectively. Different groups show a significance of $p < 0.001$ when compared to each other. #1: TDI 1 time treatment group; #20: TDI 20 times treatment group; Con: control.
